# Structural Relationships between Cognitive Achievement and Learning-Related Factors among South Korean Adolescents

**DOI:** 10.3390/jintelligence10040081

**Published:** 2022-10-09

**Authors:** Ji-Hye Lee

**Affiliations:** Department of Counseling Psychology, Seowon University, Chungbuk 28644, Korea; wisdom56@seowon.ac.kr

**Keywords:** cognitive achievement, learning-related factors, South Korea, middle school students, high school students

## Abstract

This study aimed to differentiate between middle and high school students by investigating the structural relationships between academic self-efficacy, academic stress, learning motivation, and learning strategies—the learning-related factors of South Korean adolescents’ learning achievements. We obtained relevant information for 4051 middle school students and 4242 high school students from the Gyeonggi Institute of Education’s three-year panel data study (2016–2018). The results showed direct and indirect influential relationships between academic self-efficacy, academic stress, learning motivation, and learning strategy. Results of the analysis conducted by separating the structural equation model into middle and high school student groups showed that both models met the goodness-of-fit indices criteria; the difference between middle and high school student group models was significant. Hence, to promote academic achievement among middle and high school students, differential assistance is needed. Middle school students should be assisted so that they have a good command of learning strategy after reducing academic stress by increasing academic self-efficacy. For high school students, management of academic stress by increasing academic self-efficacy is the most important aspect.

## 1. Introduction

Improvement in students’ academic achievement is one of the cognitive goals of education. Factors related to academic achievement have been investigated in various studies (Lim et al. 2016). These factors can be classified into family background, school, teacher, and student. The present study focused on student-related factors; specifically, learning-related factors were investigated to determine their influence on academic achievement. Students’ learning-related factors that have been found to significantly affect academic achievement include academic self-efficacy, learning motivation, learning style, intelligence, learning strategy, subject preference, and learning attitude (Lee 2010; Lee and Ha 2016). Lee and Ha (2016) analyzed learning-related factors that affect academic achievement through structural equation modeling using data from the Gyeonggi Education Panel Study (GEPS) of Gyeonggi province, the largest local education authority in South Korea. The data were used in view of limitations regarding generalization of statistical procedures, such as regression analysis, which do not reflect the number of participants and have led to measurement errors in previous studies.

Lee and Ha (2016), in their research on middle school students, identified academic self-efficacy among the variables affecting academic achievement, as well as learning motivation and learning strategy as exogenous and mediating variables, respectively. Results of their analysis showed the direct and indirect influential relationships of academic self-efficacy, learning motivation, and learning strategy on learning achievement. The present study expanded the work of Lee and Ha (2016) by including both middle school and high school students. As academic stress has been reported to be proportionally the highest among the various stresses that South Korean adolescents experience (Kim et al. 2009), the present study included and analyzed academic stress as a learning-related variable to shed new light on academic achievement.

Moon (2013), and Lee and Chung (2014) also established and tested a structural relationship of variables affecting academic achievement. They argued that students’ academic achievement depends on the exploration of their active participation, involvement and initiative in learning tasks, learning motivation, and emotional, academic and instrumental support from teachers and classmates. In this context, the primary purpose of this study is to establish and test a structural model between learning-related variables affecting academic achievement. 

Academic self-efficacy that is based on the concept of self-efficacy refers to a learner’s subjective confidence in learning or performing given tasks in a specific situation of learning (Kim and Park 2001). It is one of major variables as learners with higher academic self-efficacy recognize the importance of learning tasks (Yusuf 2011), and participate in learning activities proactively (Collins 1982; Bores-Rangel et al. 1990; Yusuf 2011; Schunk 1982; Schunk 2008).

Academic self-efficacy is related to learning motivation. Students with high self-efficacy tend to prefer to tackle challenging tasks than easy ones (Bores-Rangel et al. 1990; Chowdhury and Shahabuddin 2007; Yusuf 2011), make a lot of efforts to accomplish them, and remain committed to them despite difficulties. It is known that these students have less uneasiness, and employ more effective learning strategy (Locke et al. 1984). Moreover, self-efficacy is closely related to stress (Zajacova et al. 2005). Stress level varies depending on self-efficacy level. It is reported that those with high self-efficacy reduce stress by proactively addressing stressful situations (Ten Brink et al. 2021). In summary, it is assumed that academic stress, learning motivation and learning strategy are mediating variables for the relationship between academic self-efficacy and academic achievement. 

According to previous studies, academic self-efficacy significantly affected academic stress (Oh and Seon 2013) and negatively affected learning motivation (Son et al. 2015), learning strategy (Nam 2016), and academic achievement (Jo and Son 2011). In addition, a strong correlation between learning motivation and learning strategy has been verified by numerous previous studies (Black and Deci 2000; Lee 2010). In particular, intrinsic regulation motivation is positively related to cognitive strategy (Elliot and Church 1997; Pintrich 2000; Pintrich and Schrauben 1992). Hence it can be assumed that the mediating variable with the largest impact is academic stress, followed by learning motivation and learning strategy.

Accordingly, the present study examined academic stress, learning motivation, and learning strategy as mediating variables in the relationship between academic self-efficacy and learning achievement; Using a sequential and integrated approach the study attempted to identify which factors promote learning achievement in South Korean adolescents. In particular, the main purpose of the present study was to determine whether differential assistance is needed to increase learning achievement of middle and high school students. To do so, the study used structural equation modeling to determine whether a difference exists between middle and high school students. In South Korea, middle school students and high school students are adolescents, but they are in different phases of adolescence. Adolescence is usually divided into initial phase (age: 11-14), intermediate phase (14–16), and final phase (16–18), and each phase requires different tasks (Berk 2007).

In South Korea, the intermediate phase of adolescence falls in middle school period and the final phase belongs to high school period. With the school level transition, high school students receive more workloads than middle school students and face more academic stresses related to college admission (Lee and Yu 2020). Accordingly, it is important to make distinction between these two periods in analyzing a structural relationship between learning-related factors of academic achievement. The results of the present study will serve as baseline data for increasing the learning achievement of South Korean adolescents.

## 2. Methods

### 2.1. Participants

The third-year data from the 2014 GEPS were employed to analyze the structural relationships among learning-related variables that affect the learning achievement of South Korean adolescents. The data was collected from 2014 to 2016, and subsequently analyzed from August 2017 to March 2018. The third-year GEPS data included 4051 middle school and 4242 high school students.

### 2.2. Measurement Instruments

Learning achievement is considered to be cognitive learning achievement. From the GEPS data, scores obtained in Korean language, English, and mathematics were used as measures of learning achievement. To measure academic self-efficacy, participants were asked questions about their sense of efficacy in the same three areas: Korean language, English, and mathematics. To measure academic stress, related questions were classified into anxiety, relationship and competence. The GEPS item number was 15 (6 items) in 2014, 16 (6 items) in 2015, and 17 (6 items) in 2016. Examples of items were “I tend to worry about”, and “I’m not interested in everything”. To measure learning motivation, we adopted the suggestions of Lee and Ha (2016), who included only intrinsic motivation in their analysis. However, in addition to intrinsic motivation and on the basis of self-determination theory, prescribed regulation was also assessed to allow for a more meaningful analysis of learning motivation. The GEPS item number was 22 (10 items) in 2014, 23 (10 items) in 2015, and 24 (10 items) in 2016. Examples of items were “I study because I enjoy gaining knowledge”, and “I study because I like to think”. Demonstration, elaboration, organization, and meta-cognition were variables used to assess learning strategy. The GEPS item number was 23 (16 items) in 2014, 24 (16 items) in 2015, and 25 (16 items) in 2016. Examples of items were “I organize important things separately”, and “I practice by muttering over and over what I have studied”. Cronbach’s alpha coefficients for the measures were as follows: 0.82 for learning achievement, 0.82 for academic self-efficacy, 0.85 for academic stress, 0.92 for learning motivation, and 0.91 for learning strategy.

### 2.3. Data Analysis

The data analysis procedure was as follows. First, Pearson correlation coefficients were obtained using SPSS 18.0 to determine the correlations between the variables before the structural relationship analysis.

Second, goodness-of-fit indices of the structural equation model were calculated using Amos 20.0. This calculation was done to analyze structural relationships with learning-related variables that affect learning achievement.

Third, a multi-group analysis was conducted to compare the structural equation models of middle and high school student groups. In particular, goodness-of-fit indices were checked for each group by analyzing the structural equation models. Moreover, metric invariance constraints were tested by comparing regression coefficients between groups after imposing invariance constraints on the factor loading by group; the test was conducted under the assumption that measurement variables were at the same level in each group. After imposing invariance constraints on the factor loadings of the same level by group, cross-validation between the groups was interpreted to evaluate if the model’s goodness-of-fit is satisfactory. If cross-validity is established, regression coefficients between the groups can be interpreted at the same level.

Fourth, after checking if the parameters between groups were identical by testing the invariance of the final structural model, the parameter estimates and effects of each group were determined. The path coefficients were also checked to observe differences in the path coefficients of each group. If differences were found, cross-group equality constraints were sequentially performed to stringently verify whether differences were statistically significant. Specifically, observations were made to verify whether significant changes appeared in the goodness-of-fit by setting up a hierarchical model with the invariance constraints of the parameter added. If the difference in X^2^ values of the model with equality constraints of the parameter added and the model without the equality constraints was significant, then the goodness-of-fit of the model with equality constraints added was worse than that of the model without the equality constraints and that there were differences in the path coefficients between the groups.

### 2.4. Hypothetical Model

**Figure 1:**
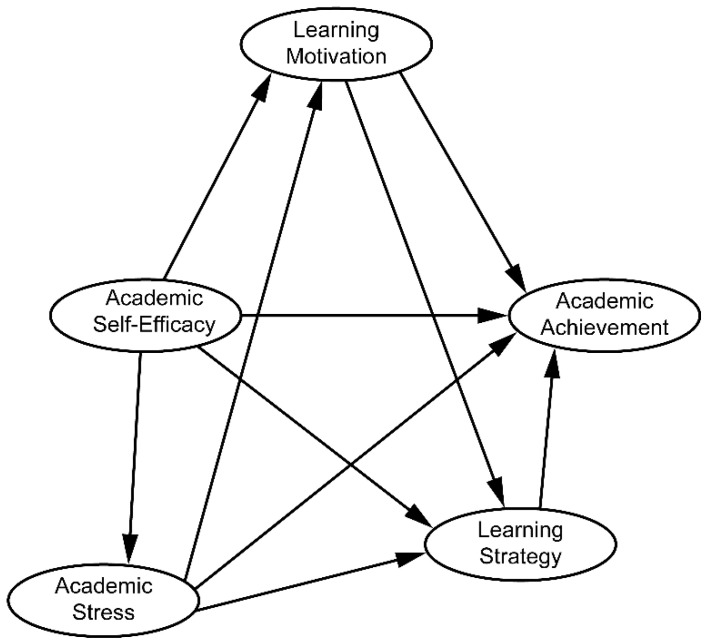
Hypothetical research model showing academic stress, learning motivation, and learning strategy as mediating variables in the relationship between academic self-efficacy and academic achievement.

The hypothetical model in Lee and Ha’s (2016) study proposed that learning motivation and strategy acted sequentially as mediating variables in the process of academic self-efficacy, which in turn helped students achieve academic success. Studies also suggest that academic self-efficacy affects academic stress (Oh and Seon 2013) learning motivation (Son et al. 2015), learning strategy (Nam 2016), and academic achievement (Jo and Son 2011). Consequently, the more positive the academic self-efficacy, the lower the academic stress, and a lower level of academic stress not only positively affects learning motivation and strategy but is also considered an important variable that increases academic achievement. As such, the hypothetical model as shown in Figure 1 was evaluated in the present study. 

### 2.5. Hypotheses

According to the academic self-efficacy theory, the academic stress theory, the learning motivation theory, the learning strategy theory, the academic achievement theory and review of the literature, the present study was designed to test the following research hypotheses.

**H1.**  *Academic self-efficacy has a direct effect on academic stress.*

**H2.**  *Academic self-efficacy has a direct effect on learning motivation.*

**H3.**  *Academic self-efficacy has a direct effect on learning strategy.*

**H4.**  *Academic self-efficacy has a direct effect on academic achievement.*

**H5.**  *Academic stress mediate the relationship between academic self-efficacy and academic achievement.*

**H6.**  *Learning motivation mediate the relationship between academic self-efficacy and academic achievement.*

**H7.**  *Learning strategy mediate the relationship between academic self-efficacy and academic achievement.*

## 3. Results

### 3.1. Correlation Analysis and Model Verification

The results of the correlation analysis showed significant correlations between academic self-efficacy, academic stress, learning motivation, learning strategy, and learning achievement, as shown in Table 1. Table 2 presents the results of the relational model on learning-related variables that affected learning achievement. The results of the goodness-of-fit test showed the indices all fit the criteria: χ^2^ = 1590.006 (*df* = 80, *ρ* = 0.000), RMSEA = 0.072, TLI = 0.935, CFI = 0.955, NFI = 0.954.

### 3.2. Analysis of Middle and High School Group Models

The structural equation models of the middle and high school student groups were separated, and the goodness-of-fit indices were obtained. The results indicated that both groups met the criteria, and the goodness-of-fit indices of the structural model with metric invariance constraints also fit the criteria (Table 3).

The satisfactory level of the goodness-of-fit of the metric invariance constraints indicated cross-validation between groups; as such, the regression coefficients between groups can be interpreted at the same level (Kim et al. 2009). Homogeneity between the groups of the models means that the degree of correlation between variables can be explained in similar ways between the groups. Table 4 shows the results. Significant differences were found in models S1 and S2; both groups were constrained by the same path coefficients in model S1, whereas both groups were constrained by not only the path coefficients but also the variance/covariance of the latent variable in model S2. In model S3, variance/covariance between path coefficients as well as the latent variable and error variance of the latent variable were constrained; these variables showed significant differences compared to the unconstrained model. Therefore, there was a significant difference between the middle and high school student group models.

### 3.3. Path Coefficients of the Structural Equation Model by Group

As discussed above, significant differences between middle and high school student group models as well as in the paths by group were observed, as shown in Table 5. Because there was a significant difference between the χ^2^ value of the model with equality constraints on every path and that of the model without equality constraints, it appears that there was a significant difference between middle and high school student group structural equation models’ paths.

**Figure 2:**
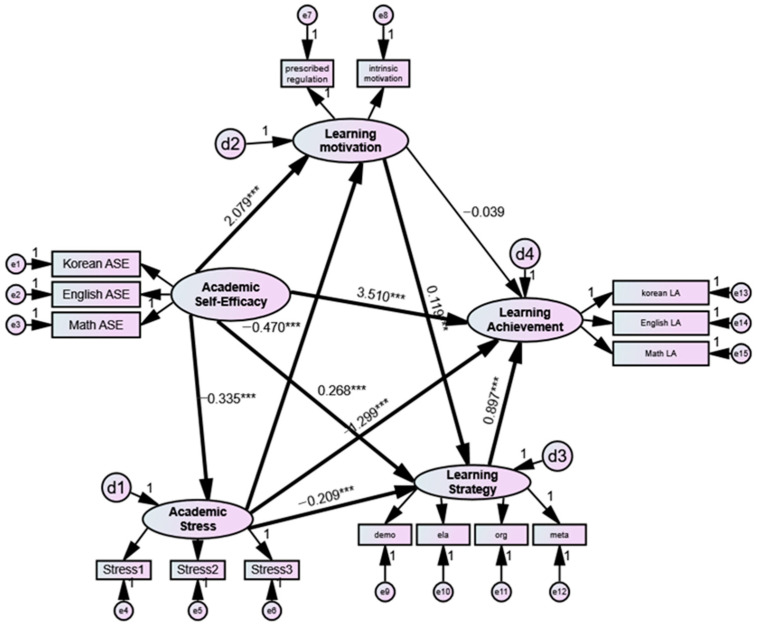
Middle school model showing academic stress, learning motivation, and learning strategy as mediating variables in the relationship between academic self-efficacy and academic achievement. **Note**: e = measurement error; d = structural error; ASE = academic self-efficacy; LA = learning achievement; stress1 = anxiety stress; stress2 = relationship stress; stress3 = competence stress; demo = demonstration; ela = elaboration; org = organization; and meta = meta-cognition. *** *p* < 0.001.

**Figure 3:**
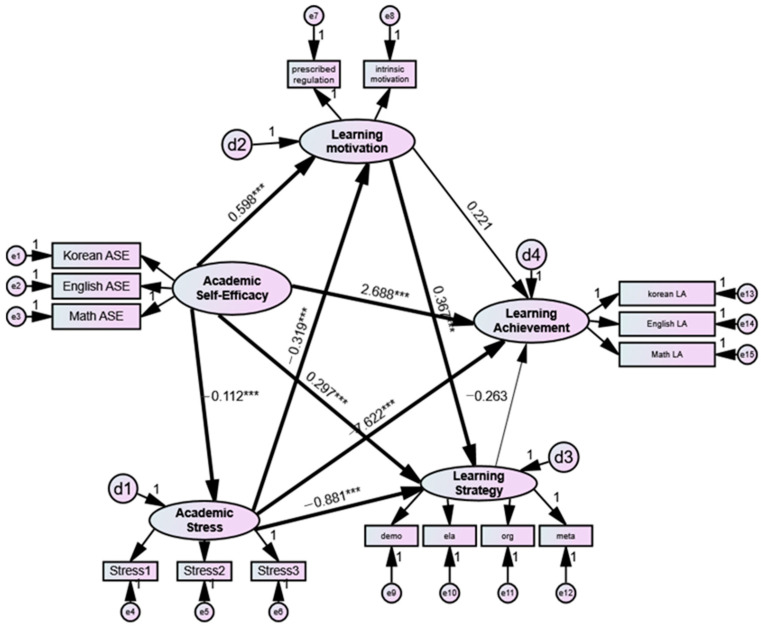
High school model showing academic stress, learning motivation, and learning strategy as mediating variables in the relationship between academic self-efficacy and academic achievement. **Note**: e = measurement error; d = structural error; ASE = academic self-efficacy; LA = learning achievement; stress1 = anxiety stress; stress2 = relationship stress; stress3 = competence stress; demo = demonstration; ela = elaboration; org = organization; and meta = meta-cognition. *** *p* < 0.001.

The results of the path coefficient analysis of each group are presented in Figure 2 and Figure 3 and Table 6. The unstandardized regression coefficients (B) were used for parameter estimation because comparison using the unstandardized regression coefficients was appropriate for determining the differences in path coefficients between groups due to the differences in variances between groups.

The results for the model of the middle school student group are shown in Figure 2. All paths except the path of learning motivation to academic achievement were statistically significant. In particular, academic self-efficacy had a significant influence on academic stress (H1 *B* = −0.335, *p* < 0.001), learning motivation (H2 *B* = 2.079, *p* < 0.001), learning strategy (H3 *B* = 0.268, *p* < 0.001), and academic achievement (H4 *B* = 3.510, *p* < 0.001). Academic stress was found to have a significant influence on learning motivation (*B* = −0.470, *p* < 0.001), learning strategy (*B* = −0.209, *p* < 0.001), and academic achievement (*B* = −1.299, *p* < 0.001). In addition, learning motivation had a significant effect on learning strategy (*B* = 0.119, *p* < 0.001), and learning strategy had a significant effect on academic achievement (*B* = 0.897, *p* < 0.001). Academic stress and learning strategy mediate the relationship between academic self-efficacy and academic achievement (H5, H7). Learning motivation does not mediate the relationship between academic self-efficacy and academic achievement (H6).

In the model for the high school student group (Figure 3), all paths showed statistical significance except the paths from learning motivation and learning strategy to academic achievement. Specifically, academic self-efficacy had a significant influence on academic stress (H1 *B* = −0.112, *p* < 0.001), learning motivation (H2 *B* = 0.598, *p* < 0.001), learning strategy (H3 *B* = 0.297, *p* < 0.001), and academic achievement (H4 *B* = 2.688, *p* < 0.001). Academic stress had a significant influence on learning motivation (*B* = −0.319, *p* < 0.001), learning strategy (*B* = −0.881, *p* < 0.001), and academic achievement (*B* = −7.622, *p* < 0.001), whereas learning motivation had a significant influence on learning strategy (*B* = 0.367, *p* < 0.001). Academic stress mediate the relationship between academic self-efficacy and academic achievement (H5). Learning motivation and learning strategy do not mediate the relationship between academic self-efficacy and academic achievement (H6, H7).

## 4. Discussion

The purpose of this study was to determine differences between middle and high school student groups by investigating structural relationships between academic self-efficacy, academic stress, learning motivation, and learning strategy—the learning-related factors of learning achievements of South Korean adolescents.

First, the structural relationships among learning-related variables that affect learning achievement of South Korean adolescents were investigated using GEPS third-year data, and the goodness-of-fit met all criteria. Consequently, the model showed both direct and indirect influential relationships among academic self-efficacy, academic stress, learning motivation, and learning strategy, which are the learning-related factors that can affect the learning achievements of South Korean adolescents. The results of the analysis—which was carried out by separating the structural equation model into middle and high school student groups—showed that both models met the goodness-of-fit indices criteria. These results indicated that the overall model investigated in the present study was appropriate for both middle and high school student groups.

Given the findings in the present study, to increase the level of academic achievement among South Korean adolescents, learning-related variables such as academic self-efficacy, academic stress, learning motivation, and learning strategy should be reviewed from many angles rather than focusing on a single variable, and approached from an integrated perspective. The results support previous studies (Hayat et al. 2020; Bong and Clark 1999; Kumar 2013; Jo and Son 2011) that showed academic self-efficacy had a large influence on the overall learning process, including low academic stress, high learning motivation, use of effective learning strategies, and improvement in academic achievement. And Hayat et al. (2020) carried out an intensive study on “Relationships between academic self-efficacy, learning-related emotions, and metacognitive learning strategies with academic performance in medical students: a structural equation model” of which the result came out as self-efficacy, metacognitive learning strategies and academic performance were significantly correlated. In addition, the results of the present study support the results of Chang (2020) which analyzed middle school students using a multivariate latent growth model and found a statistically significant path model where intrinsic regulation motivation and learning strategy are mediating variables in the relationship between self-efficacy and academic achievement. Meanwhile, the present study supports Lee and Ha (2016) which examined a structural relationship between learning-related variables affecting academic achievement of middle school students. Furthermore, the present study is consistent with the findings of the Korean Educational Longitudinal Study (Kim et al. 2013) of middle school students that there is a positive relationship between the initial values of academic achievement and academic self-efficacy, and between the variation rates of academic achievement and self-efficacy.

Second, a statistically significant difference was found between the middle and high school student groups. For the middle school students, according to the results, hypotheses H1, H2, H3, H4, H5, and H7 were all supported except for H6. In other words academic self-efficacy, academic stress, and learning strategy were found to have a significant influence on academic achievement; especially, the direct effect of academic self-efficacy was found to have the largest influence. Academic self-efficacy is one of the important factors influencing academic performance. Academic self-efficacy refers to the students’ beliefs and attitudes toward their capabilities to achieve academic success, as well as belief in their ability to fulfill academic tasks and the successful learning of the materials (Bandura 1997; Schunk and Ertmer 2020). The finding of this present study that learning motivation does not have a direct impact on academic achievement deviates from the findings of Gottfried (1990) that learning motivation is directly related to academic achievement, and from the findings of Lee (2009) that intrinsic motivation, identified motivation, and introjected motivation are positively correlated to academic records, and that extrinsic motivation and amotivation are negatively related to academic records. This may suggest that the South Korean education system focused on college admission is limited in enhancing students’ intrinsic motivation and academic performance. Ahn and Choi (2012) reported that high school students with higher extrinsic motivation had better academic achievement. In South Korea, it is not difficult to find students having high intrinsic and extrinsic motivation at schools (Lee and Shin 2012). In this regard, Moss (2010) found that students who have elevated level of intrinsic and extrinsic motivation showed outstanding performance in hypermedia educational environment. Hence, subsequent studies may need to examine the impact of extrinsic and other confirmed motivations among types of motivation proposed by the self-determination theory.

Among the high school students, according to the results, hypotheses H1, H2, H3, H4, and H5 were all supported except for H6 and H7. In other words academic self-efficacy and academic stress had a significant influence on academic achievement; and, as was found in the middle school students, the direct effect of academic self-efficacy had the largest influence. Kumari and Chamundeswari (2015) studied on “Achievement Motivation, Study Habits and Academic Achievement of Students at the Secondary Level” and found that a significant difference in the achievement motivation, study habits and academic achievement of students in different categories of schools. For South Korean students, stress related to learning is the most serious among various stresses (Jo 2004), and stress about academic records and tests is the highest (Son 2002). Students’ learning-related stress starts in the kindergarten and elementary school periods and rises as they enter middle and high schools (Bak and Park 2012). Accordingly, the level of learning-related stress soars as they go up to higher grades, which is attributed to more academic volume and burdens in tandem with higher grades (Oh and Seon 2013), preparation for admission to higher-level schools, parents’ excessive expectation, and a competitive school environment where the level of education is emphasized (Jeong and Kim 2008). Accordingly, in order to increase the academic achievement of high school students, compared with middle school students, it is more important to reduce academic stress by nurturing their academic self-efficacy.

In summary, based on the discussions and results of this study, the following conclusions are derived. 

First, a causal relationship can be established where academic self-efficacy of Korean adolescents affects academic stress, learning motivation, and learning strategy, leading to better academic achievement. Therefore, it can be understood that academic self-efficacy, academic stress, and learning motivation provide the “will” to continue learning, whereas cognitive strategies provide concrete “skills” to perform learning activities, which in turn improve academic achievement. As academic self-efficacy, academic stress, learning motivation, learning strategy, and academic achievement have a circulative relationship, academic stress may be reduced by inculcating academic self-efficacy when teaching students, and strategies to increase learning motivation should be implemented as well as teaching students learning strategies that contribute to academic achievement (Lee 2012).

Second, the consistent finding of the influence of academic self-efficacy on academic achievement in both middle and high school students highlights the need to focus on students’ self-efficacy as a way to increase academic achievement outcomes. However, there were also differences between middle school students and high school students in what contributed to academic achievement. To increase the academic achievement of South Korean middle school students, they must be able to manage academic stress by developing positive academic self-efficacy so that they can exercise effective learning strategies. Similarly, to increase high school students’ academic achievement, they must manage academic stress by developing positive academic self-efficacy; however, academic stress and self-efficacy are more important factors than learning motivation and learning strategy. According to the ‘Children’s Subjective Well-being in Rich Countries (Martorano et al. 2018)’ conducted by the United Nations Children’s Fund (UNICEF), South Korea ranked first with 50.5% of the world’s share, indicating that the academic stress of adolescents is severe. Youth in Korea are experiencing serious academic stress due to the college entrance exam system and competitive social climate. Grades are important to Korean students. Getting good grades through schooling is not everything, but in reality, grades are the most important measure for evaluating a student and a big variable in deciding on a career path (Jeong 2010). In addition, educational achievement in Korean society is recognized as an important factor that has a great influence on a student’s life as a key factor in social mobility (Kim and Koh 2007). For this reason, it can be said that tolerance to stress is more important for high school students than for middle school students. Given the current educational system’s focus on college entrance, the ability to manage academic stress is likely to be more important than learning motivation or learning strategy. 

In general, this study can be regarded as evidence regarding the direct and indirect effects of academic self-efficacy, academic stress, learning motivation, and learning strategies—the learning-related factors of South Korean adolescents’ learning achievements; it also supports the control-value theory and other studies conducted in this field. Despite these strengths, in this study, self-report questionnaires were used that raises the possibility of response bias. However, the use of self-report questionnaires enables us to elicit the participants’ beliefs and personal perceptions toward their learning process. 

## Figures and Tables

**Table 1:** Correlations among Academic Self-Efficacy, Academic Stress, Learning Motivation, Learning Strategy, and Learning Achievement.

Variable	Academic Self-Efficacy	Academic Stress	Learning Motivation	Learning Strategy
Academic stress	−0.356 **			
Learning motivation	0.459 **	−0.378 **		
Learning strategy	0.510 **	−0.452 **	0.558 **	
Learning achievement	0.436 **	−0.298 **	0.260 **	0.366 **

** *p* < 0.01.

**Table 2:** Goodness of fit of the Structural Equation Model.

Fit Index	χ^2^	RMSEA	TLI	CFI	NFI
Criteria	Higher than 0.05	Between 0.05–0.08	Higher than 0.90		
Final model	1590.006 (*df* = 80, *p* < 0.001)	0.072	0.935	0.955	0.954

Note. RMSEA = root mean square error of approximation; TLI = Tucker-Lewis index; CFI = comparative fit index; NFI = normed fit index.

**Table 3:** Middle and High School Student Group Models’ Goodness-of-fit Indices.

Fit Index	χ^2^	RMSEA	TLI	CFI	NFI
Middle school	1901.561 (*df* = 80, *p* < 0.001)	0.075	0.911	0.940	0.938
High school	1826.613 (*df* = 80, *p* < 0.001)	0.072	0.909	0.940	0.937
Measurement invariance limit	3979.582 (*df* = 170, *p* < 0.001)	0.052	0.910	0.936	0.933

Note. RMSEA = root mean square error of approximation; TLI = Tucker-Lewis index; CFI = comparative fit index; NFI = normed fit index.

**Table 4:** Final Model’s Between-Group Homogeneity Test.

Hierarchical Model		χ^2^ (*df*)	χ^d2^ (*df*Δ)	*p*
S0	Unconstrained model	3728.176 (160)	-	
S1	Same path coefficients	19,758.645 (195)	16,030.469 (35)	<0.001
S2	Path, variance/covariance of the latent variable coefficients	30,838.773 (196)	27,110.597 (36)	<0.001
S3	Path, variance/covariance, error variance of the latent variable coefficients	32,599.672 (200)	28,871.496 (40)	<0.001

**Table 5:** Equality Constraints Analysis of the Structural Equation Models of Middle and High School Student Groups.

Hierarchical Model	χ^2^ (*df*)	χ^d2^ (*df*Δ)	*p*
Unconstrained model	3728.176 (160)	-	
Academic self-efficacy → Academic stress	3765.710 (161)	37.534 (1)	0.000
Academic self-efficacy → Learning motivation	4062.051 (161)	333.875 (1)	0.000
Academic self-efficacy → Learning strategy	3729.141 (161)	0.965 (1)	0.000
Academic self-efficacy → Learning achievement	3861.848 (161)	133.672 (1)	0.000
Academic stress → Learning motivation	3738.318 (161)	10.142 (1)	0.000
Academic stress → Learning strategy	3816.356 (161)	88.18 (1)	0.000
Academic stress → Learning achievement	3736.463 (161)	8.287 (1)	0.000
Learning motivation → Learning strategy	3936.601 (161)	208.425 (1)	0.000
Learning motivation → Learning achievement	3730.758 (161)	2.582 (1)	0.000
Learning strategy → Learning achievement	3744.205 (161)	16.029 (1)	0.000

**Table 6:** Unstandardized Regression Coefficients of the Structural Equation Models of Middle and High School Student Groups.

Path	Middle School		High School	
	*B*	C.R.	*B*	C.R.
Academic self-efficacy → Academic stress	−0.335	−14.078 ***	−0.112	−16.411 ***
Academic self-efficacy → Learning motivation	2.079	25.890 ***	0.598	21.773 ***
Academic self-efficacy → Learning strategy	0.268	15.032 ***	0.297	13.510 ***
Academic self-efficacy → Learning achievement	3.510	16.300 ***	2.688	14.380 ***
Academic stress → Learning motivation	−0.470	−7.096 ***	−0.319	−4.593 ***
Academic stress → Learning strategy	−0.209	−15.367 ***	−0.881	−16.404 ***
Academic stress → Learning achievement	−1.299	−8.396 ***	−7.622	−16.405 ***
Learning motivation → Learning strategy	0.119	26.150 ***	0.367	21.984 ***
Learning motivation → Learning achievement	−0.039	−0.712	0.221	1.582
Learning strategy → Learning achievement	0.897	3.753 ***	−0.263	−1.585

Note. C.R. = critical ratio. *** *p* < 0.001.

## Data Availability

Not applicable.

## References

[B1-jintelligence-10-00081] Ahn Dohee, Choi Hyerim (2012). The relationships between academic motivation, social support, and academic achievement of high school students. Korean Education Inquiry.

[B2-jintelligence-10-00081] Bak Byunggee, Park Sunmi (2012). Development and validation of an academic stress scale. Korean Journal of Educational Psychology.

[B3-jintelligence-10-00081] Bandura Albert (1997). Self-Efficacy: The Exercise of Control.

[B4-jintelligence-10-00081] Berk Laura E. (2007). Development through the Lifespan.

[B5-jintelligence-10-00081] Black Aaron E., Deci Edward L. (2000). The effects of instructors’ autonomy support and students’ autonomous motivation on learning organic chemistry: A self-determination theory perspective. Science Education.

[B6-jintelligence-10-00081] Bong Mimi, Clark Richard E. (1999). Comparison between self-concept and self-efficacy in academic motivation research. Educational Psychologist.

[B7-jintelligence-10-00081] Bores-Rangel Enrique, Church A. Timothy, Szendre Dottie, Reeves Carolyn (1990). Reeves Self-efficacy in relation to occupational consideration and academic performance in high school equivalency students. Journal of Counseling Psychology.

[B8-jintelligence-10-00081] Chang Heesun (2020). A longitudinal study on the effect of self-efficiency on academic achievement mediated with intrinsic motivation and learning strategy. Education Evaluation Research.

[B9-jintelligence-10-00081] Chowdhury Mohammed S., Shahabuddin A. M. (2007). Self-efficacy, motivation and their relationship to academic performance of Bangladesh college students. College Quarterly.

[B10-jintelligence-10-00081] Collins Janet Lynn (1982). Self-Efficacy and Ability in Achievement Behavior.

[B11-jintelligence-10-00081] Elliot Andrew J., Church Marcy A. (1997). A hierarchical model of approach and avoidance achievement motivation. Journal of Personality and Social Psychology.

[B12-jintelligence-10-00081] Gottfried Adele E. (1990). Academic intrinsic motivation in young elementary school children. Journal of Educational Psychology.

[B13-jintelligence-10-00081] Hayat Ali Asghar, Shateri Karim, Amini Mitra, Shokrpour Nasrin (2020). Relationships between academic self-efficacy, learning-related emotions, and metacognitive learning strategies with academic performance in medical students: A structural equation model. BMC Medical Education.

[B14-jintelligence-10-00081] Jeong Eui-Sook, Kim Gab-Sook (2008). Relationship of academic stress in middle school and PITR responses. Home and Quality of Life Study.

[B15-jintelligence-10-00081] Jeong Jooyoung (2010). Analysis on the causal model between elementary school child’s academic stress and academic achievement. The Korea Educational Review.

[B16-jintelligence-10-00081] Jo Hanik, Son Seonkyung (2011). The path analysis among parents’ forced social comparison of studies, academic stress, academic self-concept, and academic achievement. Future Oriented Youth Society.

[B17-jintelligence-10-00081] Jo Minyoung (2004). A study on the influences of the stressors from school on depression in middle and high school students: Moderating effect focusing on the stress coping. Master’s thesis.

[B18-jintelligence-10-00081] Kim Ah-Yeong, Park In-Yeong (2001). Construction and validation of academic self—Efficacy scale. Korean Journal of Educational Research.

[B19-jintelligence-10-00081] Kim Joohwan, Kim Mingyu, Hong Sehee (2009). Writing Thesis with Structural Equation.

[B20-jintelligence-10-00081] Kim Seonsook, Koh Misun (2007). The factor of effect in growth of academic achievement in adolescent: The use of latent growth model. Korean Youth Research.

[B21-jintelligence-10-00081] Kim Yangboon, Lim Hyeonjeong, Namgung Jiyoung, Park Heejin, Shin Hyesook, Kim Seongsik, Kim Jongmin, Lee Kyumin, Ban Jaechun (2013). Longitudinal Study on Korean Education.

[B22-jintelligence-10-00081] Kumari Archana, Chamundeswari S. (2015). Achievement motivation, study habits and academic achievement of students at the secondary level. International Journal of Emerging Research in Management &Technology.

[B23-jintelligence-10-00081] Kumar Dinesh (2013). Academic achievement of secondary school students in relation to academic motivation. Research Analysis and Evaluation.

[B24-jintelligence-10-00081] Lee Eunjee, Yu Jihye (2020). The analysis of the longitudinal causal relationship among academic achievement, school adaptation, and life satisfaction of adolescents in high school transition. The Journal of Learner-Centered Curriculum and Instruction.

[B25-jintelligence-10-00081] Lee Jihye (2010). Analysis of the structural relationships among self-determination motivation to learn, metacognition, self-directed learning ability, learning flow, and school achievement. Korean Journal of Educational Research.

[B26-jintelligence-10-00081] Lee Jihye, Ha Jungyoon (2016). Effects of academic self-efficacy, learning motivation, and learning strategy on academic achievement: Comparison of instructor-centered instruction and learner-centered instruction. The Journal of Learner-Centered Curriculum and Instruction.

[B27-jintelligence-10-00081] Lee Jungsoo, Chung Youngran (2014). An analysis of structural relationship among the attitude toward science, science motivation, self-regulated learning strategy, and science achievement in middle school students. Journal of the Korean Association for Science Education.

[B28-jintelligence-10-00081] Lee Minhee (2009). Counseling strategies to improve adolescents’ academic performance based on self-determination theory. Korean Journal of Counseling and Psychotherapy.

[B29-jintelligence-10-00081] Lee Sujin (2012). The relationship of academic self-efficacy, task value, achievement goal, academic efforts and cognitive strategies to English academic achievement between middle and high school students in Korea. The Journal of Educational Research.

[B30-jintelligence-10-00081] Lee Sujin, Shin Hyunjoo (2012). The effect of academic motivation, academic efforts and self-regulation learning strategies on English academic achievement between middle and high school students in Korea. Secondary Education Research.

[B31-jintelligence-10-00081] Lim Hyunjung, Si Gija, Kim Seongeun (2016). Student achievement trends and related factors the analysis of NAEA longitudinal data. Journal of Educational Evaluation.

[B32-jintelligence-10-00081] Locke Edwin A., Frederick Elizabeth, Lee Cynthia, Bobko Philip (1984). Effect of self-efficacy, goals, and task strategies on task performance. Journal of Applied Psychology.

[B33-jintelligence-10-00081] Martorano Bruno, Natali Luisa, Neubourg Chris De, Bradshaw Jonathan (2018). Children’s Subjective Well-being in Rich Countries.

[B34-jintelligence-10-00081] Moon Ehunshik (2013). The structural relationship among the classroom social environment, motivational beliefs, engagement, and academic achievement in middle school students. The Korean Journal of Child Education.

[B35-jintelligence-10-00081] Moss Hans-Martin (2010). Influence of Stakeholder Motivation on the Outcome of IT Projects.

[B36-jintelligence-10-00081] Nam Yoongon (2016). Relationship between Academic Stress and Self-Regulated Learning Strategies: Focusing on Mediating Effect of Academic Motivation. Master’s thesis.

[B37-jintelligence-10-00081] Oh Jeonghee, Seon Hyeyeon (2013). The Study on the factors related to Academic stress of Elementary and Middle School Students. Korean Journal of Counseling.

[B38-jintelligence-10-00081] Pintrich Paul R., Boekaerts M., Pintrich P. R., Zeidner M. (2000). The role of goal orientation in self-regulated learning. Handbook of Self-Regulation.

[B39-jintelligence-10-00081] Pintrich Paul R., Schrauben Barbara, Schunk Dale H., Meece Judith L. (1992). Students’ motivational beliefs and their cognitive engagement in classroom academic tasks. Student Perceptions in the Classroom.

[B40-jintelligence-10-00081] Schunk Dale H. (1982). Effects of effort attributional feedback on children’s perceived self-efficacy and achievement. Journal of Educational Psychology.

[B41-jintelligence-10-00081] Schunk Dale H. (2008). Self-efficacy, motivation, and performance. Journal of Applied Sport Psychology.

[B42-jintelligence-10-00081] Schunk Dale H., Ertmer Peggy A., Boekaerts Monique, Pintrich Paul R., Zeidner Moshe (2020). Self-regulation and academic learning: Self-efficacy enhancing interventions. Handbook of Self-Regulation.

[B43-jintelligence-10-00081] Son Changsuk, Lee Jumi, Lee Myungsook (2015). The structural relationship among academic stress, learning motivation, and emotion regulation of elementary school students. Teacher Education Research.

[B44-jintelligence-10-00081] Son Hyangsuk (2002). A study on stress factor and coping behavior of high school student. Master’s thesis.

[B45-jintelligence-10-00081] Ten Brink Maia, Lee Hae Yeon, Manber Rachel, Yeager David S., Gross James J. (2021). Stress, sleep, and coping self-efficacy in adolescents. Journal of Youth and Adolescence.

[B46-jintelligence-10-00081] Yusuf Muhammed (2011). The impact of self-efficacy, achievement motivation, and self-regulated learning strategies on students’ academic achievement. Procedia—Social and Behavioral Sciences.

[B47-jintelligence-10-00081] Zajacova Anna, Lynch Scott M., Espenshade Thomas J. (2005). Self-efficacy, stress, and academic success in college. Research in Higher Education.

